# Care in Context: Dementia Support in Mexico and the United States

**DOI:** 10.1093/geroni/igab046.1841

**Published:** 2021-12-17

**Authors:** Sunshine Rote, Jacqueline Angel, William Vega

## Abstract

Due to rapid demographic transitions, the number of people with dementia is rising in the Americas, and is expected to double in the coming decades, increasing from14.8 million in 2030 to over 27 million by 2050. The burden of dementia is especially pronounced for the Mexican-origin population in Mexico and the U.S. For Mexico, financial support for older low-income citizens and medical care are universal rights, but limited fiscal resources and the needs of a large low-income population create inevitable competition for limited resources among the old and the young. Although the United States has a more developed economy and well-developed Social Security and health care financing systems for older adults, Mexican-origin individuals in the U.S. do not necessarily benefit fully from these programs. The institutional and financial problems are compounded in both countries by longer life spans, smaller families, as well as changing gender roles and cultural norms. Such changes affect the Mexican-origin population in particular because of a higher prevalence rates of cognitive impairment than other racial and ethnic groups, and the lower access to resources to provide care. In this GSA Symposium, the authors of four papers deal with the following topics as they relate to dementia care in Mexico and the United States: (1) living alone in late life; (2) living arrangements and dementia care; (3) the role of non-governmental organizations in care; (4) next steps to address dementia care needs in the U.S. and Mexico.

